# Influence of ZrO_2_ content on the mechanical, electrical, and microstructural characteristics of La_1-*x*_Zr_*x*_Co_1−y_Mn_y_O_3_ perovskites for IT-SOFC cathodes

**DOI:** 10.1371/journal.pone.0320562

**Published:** 2025-06-04

**Authors:** Md. Abu Daud, Robel Ahmed, Md. Nurul Islam, Md Mahadi Hassan Parvez, Md. Shofiqul Islam, M. A. Gafur, Aninda Nafis Ahmed

**Affiliations:** 1 Bangladesh Army University of Science and Technology (BAUST), Department of Mechanical Engineering, Saidpur Cantonment, Saidpur, Nilphamari, Bangladesh; 2 Rajshahi University of Engineering & Technology (RUET), Department of Mechanical Engineering, Rajshahi, Bangladesh; 3 Pilot Plant & Process Development Centre (PP & PDC), Bangladesh Council of Scientific and Industrial Research (BCSIR), Dhaka, Bangladesh; Ural Federal University, Ural Power Engineering Institute, RUSSIAN FEDERATION

## Abstract

In this research, the doping effects of ZrO_2_ and MnO_2_ on La_1-*x*_Zr_*x*_Co_1−y_Mn_y_O_3_ cathode were investigated in terms of physical, mechanical and electrical properties. The amount of ZrO_2_ was varied by 5wt%, 10wt%, and 15wt% for different compositions of the composites and MnO_2_ was varied accordingly. The composite cathode is prepared to enhance the structural and functional properties of La_1-*x*_Zr_*x*_Co_1−y_Mn_y_O_3_ composites by varying ZrO_2_ doping levels, optimizing their suitability for high-performance applications through detailed material characterization in powder and pellet form, followed by calcination at 1000°C and sintering at 1200°C. The final sintered composites were then examined by SEM-EDX, XRD, and AFM. Investigations were also conducted on density, porosity, compressive strength, thermal expansion coefficient (TEC), electronic conductivity, and diametral tensile strength (DTS). SEM and EDX shows both imaging and chemical analysis of the composites which indicates the results of reactions during sintering. XRD indicates that significant structural change had been taken place with the addition of ZrO_2_. These defects in perovskite structure will increase the ionic and electronic conductivity of the composites. The highest value of DTS, compressive strength was obtained for 15LZCM sample and lowest value of DTS, and compressive strength was observed for the 5LZCM sample. Some properties like microhardness, thermal expansion, and electrical conductivity were also determined. XRD analysis shows ZrO_2_ doping caused transformation of the perovskite structure and the leading crystal system was monoclinic (P 1 21/c1). SEM shows the porous microstructure of the perovskite oxide. AFM reveals the addition of the ZrO_2_ decreasing roughness; the rms roughness of 5LZCM was 61.46 nm but the rms roughness was 37.12 nm for 15LZCM.

## Introduction

Intermediate-temperature solid oxide fuel cell (IT-SOFC) systems generally use solid ceramics as electrolytes and operate at very high temperatures (600–1000°C). This high operating temperature enables rapid electro-catalytic reactions with base metals, enabling internal reforming and producing good-quality by-product heat for cogeneration. This fuel cell has an efficiency potential of up to 70% and a further 20% potential for heat recovery [1,2]. Due to the long duration needed to attain operational temperatures, SOFCs are most appropriate for ensuring the supply of electricity in utility applications [3,4]. Fuel cells work as long as fuel and oxidant are supplied to the electrodes and the environmental impact is negligible. There are several fuel cell kinds, and they are often categorized by the chemical properties of the electrolyte utilized as the cell’s ionic conductor such as Phosphoric Acid (PAFC), Alkaline (AFC), Proton Exchange Membrane (PMFC), Direct Methanol (DMFC), Sulfuric Acid (SAFC), Solid Oxide (SOFC), Molten Carbonate (MCFC), and Protonic Ceramic Fuel Cell (PCFC) [1,5,6]. Perovskite compounds with the general formula ABO_3_, where A stands for alkaline-earth or rare-earth metal cations and B is transition-metal cations, have gained considerable attention as a type of typical cathode materials to their compositional and structural flexibility, greater oxygen reduction reaction activity, simplicity of access, and low environmental impact [7].

In comparison to cobalt-based materials, iron-based SOFC materials have a higher combined electronic and ionic conductivity as well as a low price and thermal expansion coefficient [8,9] Moreover, the majority of the iron-based perovskites produced so far even have poor chemical and structural stability against the surrounding atmosphere and poorer electro-catalytic activity at low temperatures, which severely limits their prospective uses [3,10,11]. A parent chemical BiFeO_3_ is considered to be one of the best cathode possibilities for SOFC [8,11]. Bi_2_Fe_4_O_9_ and Bi_20_FeO_40_ are examples of impurity phases that repeatedly develop throughout the synthesis process, and bismuth’s high volatility is one of the main barriers to SOFC usage of BiFeO_3_ [12]. Dopants play a crucial role in enhancing material properties, particularly in shielding glasses [13]. For instance, adding CuO to B_2_O_3_-Li_2_O-Na_2_O-CaO-SrO-As_2_O_3_ glass significantly improves its optical and structural characteristics [14]. This enhancement is further evidenced by the increased absorption of neutrons and photons, making such doped glasses highly effective for radiation shielding applications. Because of the lesser valence of Ba^2+^ over Bi^3+^ (charge compensation), the doping of Ba^2+^ considerably inhibited the secondary phase. It encouraged the production of oxygen vacancies, resulting in the successful preparation of a single-phased Bi_1-x_Ba_x_FeO_3_ [15]. Based on the fact that SrFeO_3_ is a mixed conductor, the Ba_0.5_Sr_0.5_Zn_0.2_Fe_0.8_O_3_ (BSZF) perovskite oxide was created as a new cobalt-free oxygen porous membrane [16,17]. At room temperature, the addition of copper ions to BaFeO_3__–__𝛿_ (BF) partially substituted the B-site ions with oxygen vacancies, stabilizing the cubic structure. The result was a membrane with increased oxygen permeability compared to the original BF membrane. The findings indicate that Cu doped BF has the potential to be a suitable cathode material for IT-SOFC, since it exhibits strong electrochemical performance [18]. The electro catalytic performance of cubic BF is exceptional, however achieving the synthesis of the cubic BF perovskite phase at low temperatures is very challenging [6,19]. Doping strategies in perovskite oxides, such as Ba^2+^ in BiFeO_3_, SrFeO_3_-based BSZF, and Cu in BaFeO_3_, enhance oxygen vacancies, stabilize structures, and improve oxygen permeability and electrochemical performance, making them promising for energy-related applications like IT-SOFC cathodes.

La_1-x_Sr_x_MnO_3-y_ (LSM) was the preferred material due to its strong electrical conductivity (200−300 S/cm at 900°C). The ionic conductivity, however, is quite small (10^−7^ S/cm at 900°C). In contrast, La_1-x_Sr_x_FeO_3-y_ (LSF) offers a balanced combination of electronic and ionic conduction, making it a promising alternative for IT-SOFCs [20]. Further extending the potential for high-performance applications, La_1-x_Sr_x_CoO_3-y_ (LSC) exhibits exceptional electrical conductivity of up to 1,600 S/cm at 800°C, making it particularly suitable for advanced systems [21]. Ba_1-x_Sr_x_Co_y_Fe_1-y_O_3-y_ (BSCF) has been used as a component for oxygen-separating membranes due to its excellent oxygen surface diffusion and exchange capabilities until it was further suggested as an SOFC cathode material [22]. Perovskites containing Ni and Mn elements have been extensively studied as possible cathodes in solid oxide fuel cells (SOFCs) due to the creation of oxygen vacancies in the lattice and the multivalent nature of these metals. The physical and electrochemical properties of double perovskite materials are widely acknowledged to be greatly influenced by the valence state of the cation and the presence of oxygen vacancies [23]. It was observed that if LaCo_2_O_4_ is doped with MnO_2_, Oxygen ion conductivity is increased along with its sintering capability, and density as demonstrated by Richter et al. [24] and the addition of ZrO_2_, which can lower the sintering temperature and increase the size of the grain. It can also increase the density and lower the porosity as described by Muller et al [25]. The weight percentage of MnO_2_ was kept constant and ZrO_2_ was varied to observe the impacts of ZrO_2_ on properties of MnO_2_-doped LaCo_2_O_4_. Factors with cobalt-based cathodes include incompatibility with zirconia-based electrolytes, low chemical stability, facile cobalt evaporation and reduction, and a high price for the element itself [26,27].

The previous studies highlight advancements in doping strategies for perovskite oxides to improve stability, oxygen vacancies, and electrochemical performance. In this work a new ceramic composite material was fabricated that will act as a cathode material of SOFC having improved properties, i.e., improving sintering aid, increased hardness, higher electrical conductivity, lower coefficient of thermal expansion, mechanical strength, etc.

This research focuses on the modification of perovskite oxides (La_1-*x*_Zr_*x*_Co_1−y_Mn_y_O_3_) by systematically varying ZrO_2_ doping as A-site substitution while maintaining MnO_2_ as constant B-site substitution. This study examines the simultaneous influences on structural, mechanical, thermal, and electrical properties. The study utilizes advanced characterization techniques to investigate the effects of ZrO_2_ doping on crystal structure transitions, porosity, and functional properties, providing insights for the optimization of perovskite composites in high-performance energy for IT-SOFC applications.

## Materials and methods

### 2.1 Materials

The raw materials that were used in this research are Co_2_O_3_, MnO_2_, La_2_O_3,_ and ZrO_2_. The raw materials were collected from Sisco Research Laboratories Pvt. Ltd.; India. 99% pure Zirconium oxide (ZrO_2_), Manganese dioxide (MnO_2_), and Cobalt oxide (Co_2_O_3_) were used with grain sizes of 100–200 nm, 50–100 nm, and 10 μm respectively.

### 2.2 Method

Four batches of composite pellets as listed in Table 1 were fabricated, each containing a different weight percentage of the raw materials (ZrO_2_ and MnO_2_). During the sample preparation, 5wt%, 10wt%, and 15wt% of ZrO_2_ and 30wt% of MnO_2_ were kept, and then the rest of the weight percentage was filled by the other two raw materials (Co_2_O_3_, La_2_O_3_) out of the 40 gm of the total weight of each batch. ANDGULF analytical balance (Division: 0.01 gm) was used for measuring weight for sample preparation.

**Table 1:** The weight percentage of ZrO_2_ and MnO_2_ in the fabricated composite.

Fabricated Composites	wt% of ZrO_2_	wt% of MnO_2_
5LZCM	5	30
10LZCM	10	30
15LZCM	15	30

**Fig 1:**
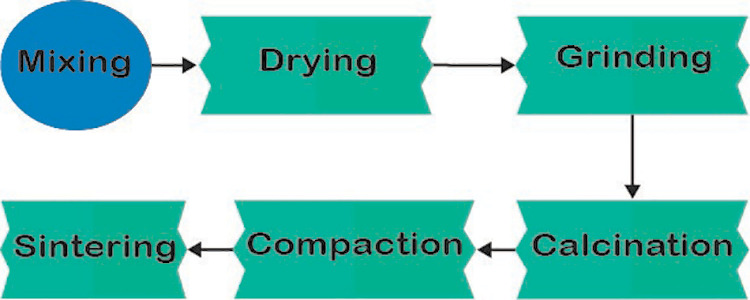
Different steps of the fabrication process.

The fabrication process was carried out by the solid-state reaction method which is commonly used for preparing polycrystalline solid from a combination of solid starting materials. The fabrication process is shown in Fig 1.

After weighing the constituents according to the proposition, all the instruments responsible for the mixing process were cleaned properly using detergent, distilled water, and ethanol. The powder mixing process was carried out by the wet ball milling process that was carried out by a pot mill (Model: G91, rpm:100). Milling was continued for 20 hours using Yttria (Y_2_O_3_) Stabilized Zirconia balls as grinding media and ethanol was used as a wetting agent. The drying process was carried out by putting a beaker in the drying oven (Manufacturer: Jisico, Model: VARO/8P) on account for ethanol to be evaporated for 24 hours at 100^0^C temperature. The grinding process was used to break the agglomerated powder using mortar pestle that continued until the fine powder was obtained.

The calcination, i.e., a purification process was carried out for 90 minutes followed by the furnace holding at 1000°C with a 5°C/min heating rate. Calcination was used before compaction to reduce impurities and to provide better compaction of the powders to form the green bodies.

**Fig 2:**
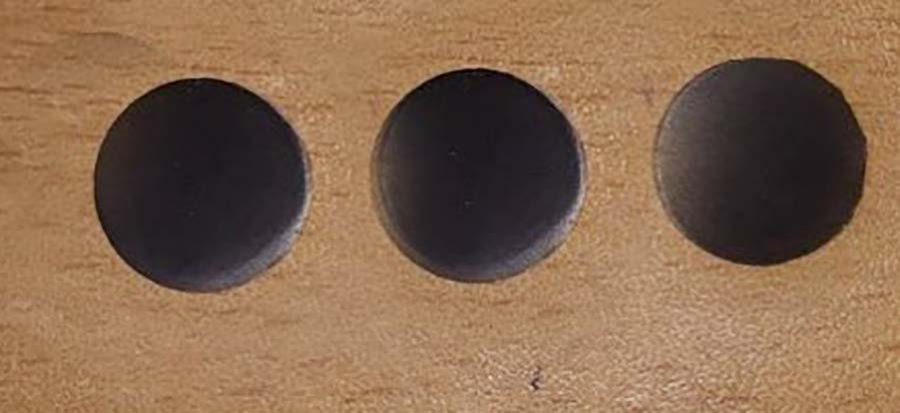
Fabricated Composites.

1.5 gm of composite sample was pressed through a pellet press (Manufacturer: Rectsch; Model: PP25) to form the green bodies where 250 MPa pressure was applied for 3 min. Sintering was completed in a sintering furnace (Manufacturer: Nabertherm, Germany; Maximum heating temperature: 1600°C) at a temperature of 1200°C. The samples were heated to 1200°C with a uniform heating rate of 5°C/min for 250 minutes and then kept at 1200°C for 240 minutes followed by furnace cooling rate for 420 minutes. The desired final composites (Fig 2) were obtained after sintering.

### 2.3 Characterization

The bulk density and porosity of the final composites were measured using the Archimedes Principle. Diametral Tensile Strength (DTS) was determined using a servo-hydraulic type Universal Testing Machine (Manufacturer: Shenzhen Wance Testing Machine Co. Ltd., China; Model: HUT 106) following ASTM D8289. The hardness test was carried out using Vicker’s hardness tester (Manufacturer: Struers, Japan; Model: DK-2) followed by ASTM E384 where the applied load should be from 1 gf to 1000 gf for the micro-Vickers test. A resistance measuring setup was developed to determine the electrical conductivity. A thermal expansion measuring setup was developed to measure the coefficient of thermal expansion (CTE). The temperature was obtained by using an inductor heater.

Scanning electron microscope (SEM: TESCAN VEGA COMPACT, Czech Republic) was utilized to monitor the microstructures of the as sintered surfaces. Energy Dispersive X-ray (EDX) was employed to carry out compositional elemental analysis of the sintered pellets using TESCAN Essence™. SEM-EDX presents information about surface morphology and elemental composition.

Crystallographic structure analysis of sintered pellets was performed using XRD (PANalytical EMP 3 X-Ray Diffractometer system, Netherlands) utilizing a Cu X-ray source (wavelength: Kα_1 _= 1.54060A˚). HighScore Plus software was used to carry out Rietveld refining.

Atomic force microscopy (AFM) studies were carried out using Nano Observer, CSI, France. Gwyddion 2.65 SPM (AFM) Software was used to analyze the images. AFM provides visualization of surface topography at the nanoscale, facilitating quantitative analysis of roughness, peaks, and pits for the evaluation of microstructural properties.

## Results and discussions

### 3.1 X-ray diffraction (XRD)

**Fig 3:**
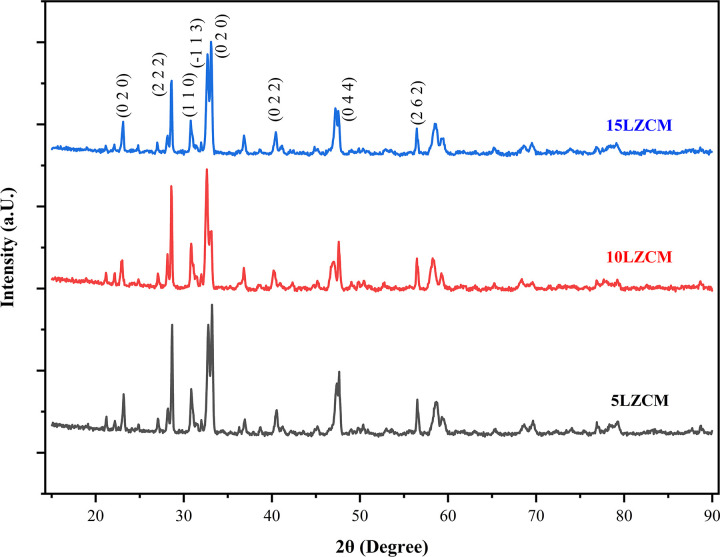
XRD pattern of the fabricated LZCM composites.

The XRD pattern is given in Fig 3 which shows the maximum peak was obtained for 2θ = 33.10° also the pattern indicates that was significant structural change has taken place with the addition of ZrO_2_. There were four types of compounds were obtained for 5LZCM and 10LZCM. For 5LZCM, the leading crystal system was found monoclinic and space group was P 1 21/c1. The structure of 5LZCM was changed from cubic (F d −3 m) to monoclinic (P 1 21/c1) or orthorhombic (P n m a) or orthorhombic (P n m a). For 10LCZM, also the crystal system was obtained monoclinic and space group was P 1 21/c1 and the crystal system was changed from cubic (F d −3 m) to monoclinic (P 1 21/c1) or monoclinic (C 12/m1) or orthorhombic (P n m a) or cubic (I a-3). For 15LZCM, four crystal system was found and the leading crystal system was monoclinic (P 1 21/c1). The crystal system was changed from monoclinic (P 1 21/c1) to cubic (F d −3 m) or cubic (F d −3 m) or orthorhombic (P n m a). The crystal system mostly relies on the type of A-site and B-site doping of the perovskite [28]. The data was collected by the Rietveld refinement using Highscrore plus software.

Table 2 highlights the unit cell parameters and crystal structures for different LZCM composites. For 5LZCM, the crystal system transitions from cubic (F d −3 m) to monoclinic (P 1 21/c1) and orthorhombic (P n m a). In 10LZCM, the crystal structure transitions from cubic (F d −3 m) to monoclinic (P 1 21/c1), orthorhombic (P n m a), and cubic (I a-3). The monoclinic (P 1 21/c1) system is prevalent in 15LZCM, with cubic and orthorhombic phases being seen. The table provides the unit cell properties, including lattice constants and volume, for each crystal system.

**Table 2:** Obtained parameters of unit cell for the different crystal systems of the fabricated composites.

Fabricated Composites	Crystal System (Space Group)	Parameters of unit cell
**5LZCM**	Cubic (F d −3 m)	a = b = c = 10.80 Å
		α = β = γ = 90°
		V = 1262.51 × 10^6^ pm^3^
	Monoclinic (P 1 21/c1)	a = 5.50 Å b = 5.43 Å c = 9.49 Å
	59.6%	α = γ = 90° β = 125.41°
		V = 231.76 × 10^6^ pm^3^
	Orthorhombic (P n m a)	a = 5.55 Å b = 7.97 Å c = 5.57 Å
		α = β = γ = 90°
		V = 246.71 × 10^6^ pm^3^
	Orthorhombic (P n m a)	a = 5.70 Å b = 7.69 Å c = 5.53 Å
		α = β = γ = 90°
		V = 242.81 × 10^6^ pm^3^
**10LZCM**	Cubic (F d −3 m)	a = b = c = 10.81 Å
		α = β = γ = 90°
		V = 1262.51 × 10^6^ pm^3^
	Monoclinic (P 1 21/c1)	a = 5.51 Å b = 5.44 Å c = 9.49 Å
	53.2%	α = γ = 90°; β = 125.41
		V = 231.70 × 10^6^ pm^3^
	Orthorhombic (P n m a)	a = 5.70 Å b = 7.69 Å c = 5.53 Å
		α = β = γ = 90°
		V = 242.81 × 10^6^ pm^3^
	Cubic (I a-3)	a = b = c = 11.39 Å
		α = β = γ = 90°
		V = 1478.43 × 10^6^ pm^3^
**15LZCM**	Monoclinic (P 1 21/c1)	a = 5.50 Å b = 5.43 Å c = 9.49 Å
	73.4%	α = γ = 90° β = 125.41
		V = 231.70 × 10^6^ pm^3^
	Cubic (F d −3 m)	a = b = c = 10.81 Å
		α = β = γ = 90°
		V = 1262.51 × 10^6^ pm^3^
	Cubic (F d −3 m)	a = b = c = 10.81 Å
		α = β = γ = 90°
		V = 1262.51 × 10^6^ pm^3^
	Orthorhombic (P n m a)	a = 5.74 Å b = 7.69 Å c = 5.53 Å
		α = β = γ = 90°
		V = 244.80 × 10^6^ pm^3^

### 3.2 Diametral tensile strength (DTS)

**Fig 4:**
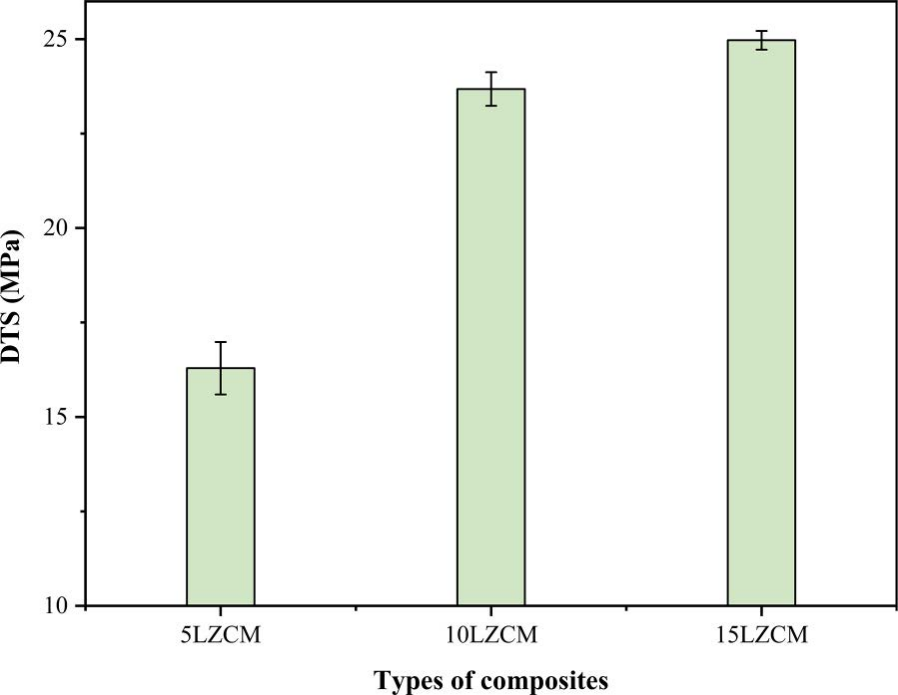
Effect of the addition of ZrO_2_ on DTS of the perovskite structure.

Fig 4 shows that DTS was increased with the increase of doping material of smaller size than the core material of the perovskite structure. It is because the smaller Zr replaces the larger La and Mn replaces the Co. From Fig 4, it was observed that the diametral tensile strength is increased from 16.29 MPa to 24.97 MPa as doping concentration increases from 5LZCM to 15LZCM. From the previous result, it was clear that DTS increased as the density increased [29]. But DTS was decreased with the increase of porosity. Hardness and DTS are opposite to the density and porosity of the composites. Till the 15LZCM, the critical point was not found where the DTS would start to decrease due to more internal defects in the structure.

### 3.3 Compressive strength (rectangular specimen)

**Fig 5:**
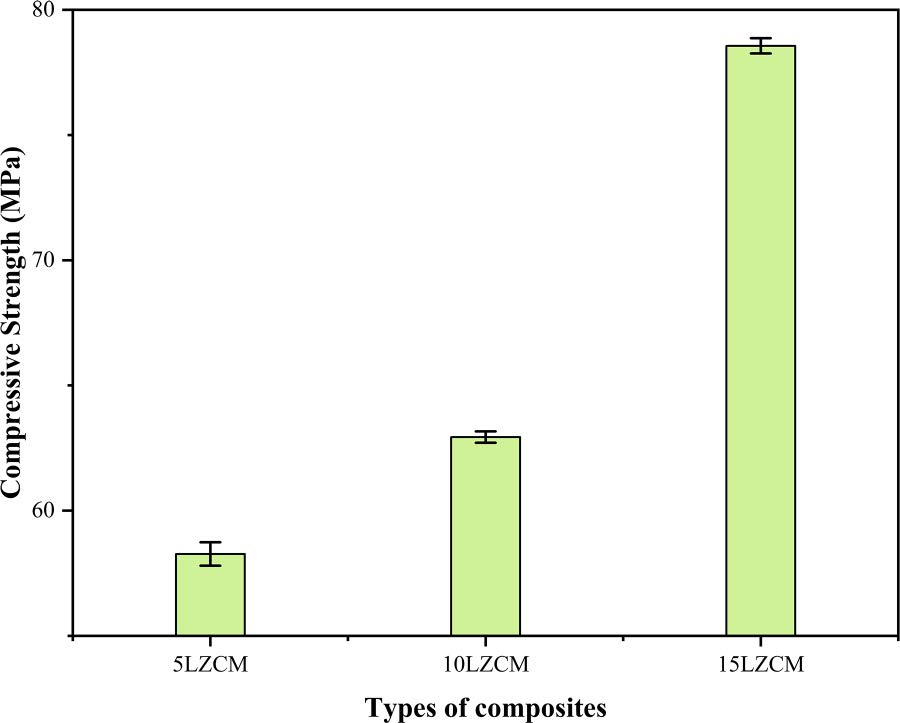
Effect of addition of compressive strength of perovskite structure.

Fig 5 shows that with the increase of doping material of smaller size than the core material of perovskite structure, compressive strength was increased. It is because the smaller Zr replaces the larger La and Mn replaces the Co. From the figure, it was observed that the compressive strength is increased from 58.27MPa to 78.56MPa as the doping concentration increases from 5LZCM to 15LZCM. Compressive strength was increased as the density increased. But compressive strength was decreased with the increase of porosity [30,31].

### 3.4 Hardness

**Fig 6:**
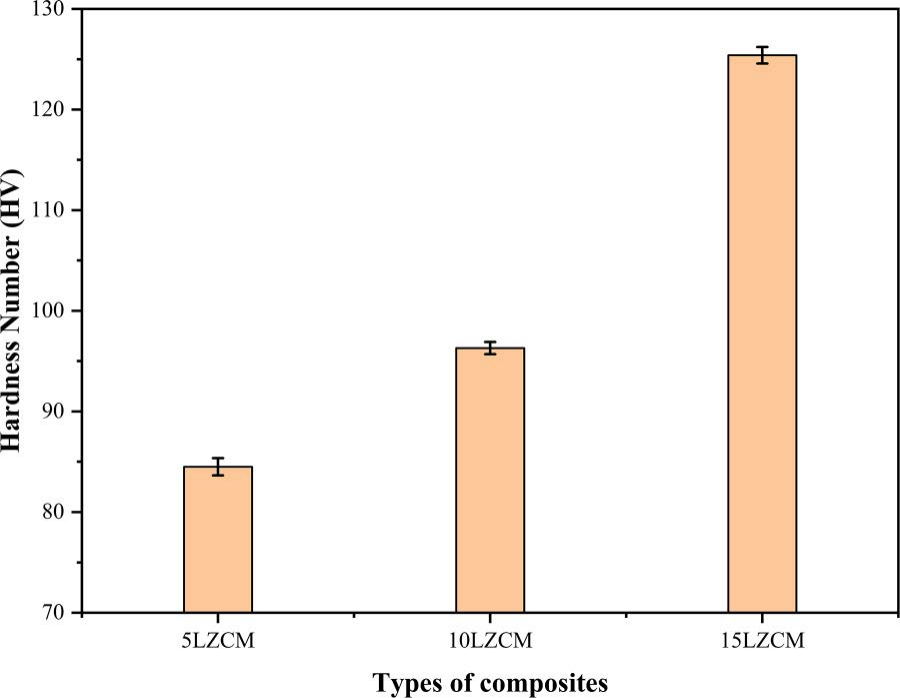
Effect of addition of ZrO_2_ on Hardness of perovskite structure.

The effect of ZrO_2_ addition on the hardness of Perovskite composites is shown in Fig 6. The hardness increased with the increment of the addition of doping material as ZrO_2_ substitute A site cation and MnO_2_ substitute B site cation of perovskite structure and the hardness increased gradually from 84.5 HV to 125.4 HV. The hardness will decrease at some point with further addition of ZrO_2_. Vickers hardness is related to the density and percentage of porosity. The hardness is maximum at that point where the density is maximum and porosity is minimum [32].

### 3.5 Coefficient of thermal expansion (CTE)

**Fig 7:**
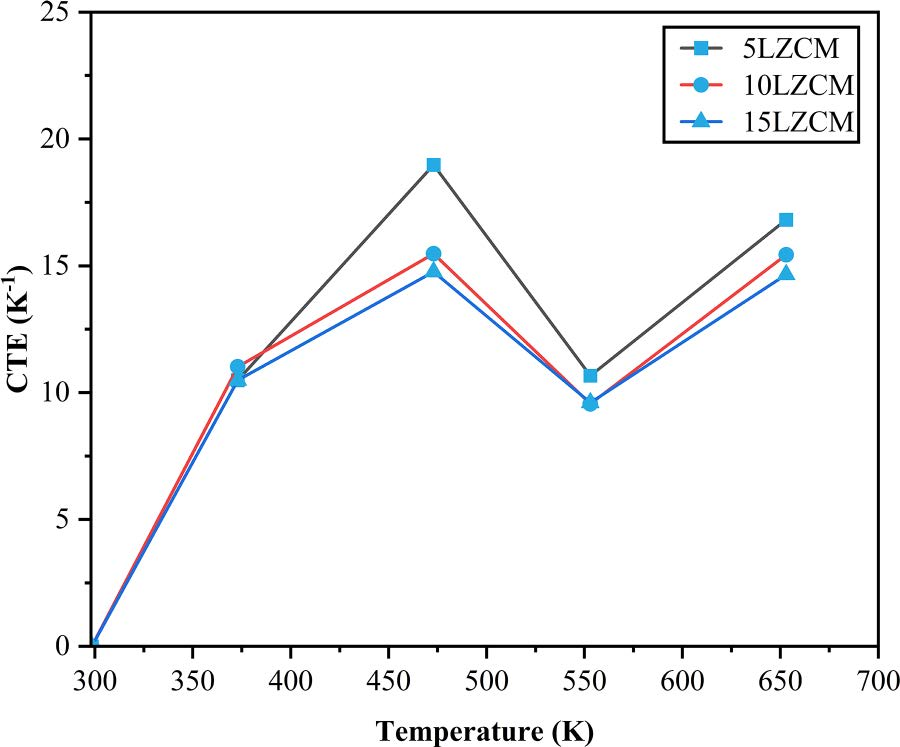
Effect of ZrO_2_ Addition on CTE.

The effect of ZrO_2_ addition on the thermal expansion coefficient is shown in Fig 7 where the sintering temperature of composites was 1200°C and the ZrO_2_ content varied from 5 wt% to 15 wt% as A site substitution, and MnO_2_ content was kept at 30 wt% as B site substitution. The figure shows that CTE decreases with the addition of ZrO_2_. The minimum CTE of the composite was obtained for 15LZCM. The CTE decreased since the nanoparticles of ZrO_2_ filled the pores of composites. The grain size of ZrO_2_ is smaller than the La_2_O_3_ thus increasing the CTE and with increasing La_2_O_3_ increasing CTE [33].

### 3.6 Electrical conductivity (σ)

**Fig 8:**
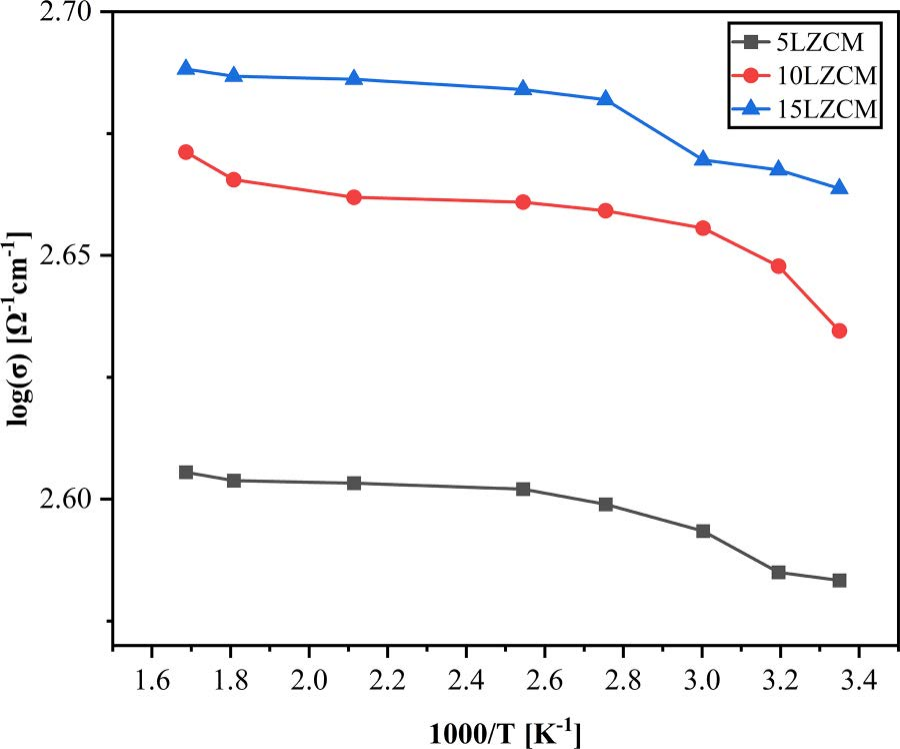
Effect of ZrO_2_ addition on electrical conductivity.

Fig 8 shows the effect of ZrO_2_ addition on the electrical conductivity. The figure shows that the electrical conductivity increases with the addition of ZrO_2_ and MnO_2_ content. The electrical conductivity increases since the nanoparticles of ZrO_2_ fill the pores of composites. The grain size of ZrO_2_ is smaller than La_2_O_3_. Thus, increasing the intrinsic defects on the lattice structure and increasing the electrical conductivity. When a smaller Zr cation replaces the larger La as A site substitution, the perfect cubic structure of base composite material LaCoO_3_ is modified so that the intrinsic defects allow the electron to pass through the material easily. Zr as divalent acceptor substitution for the trivalent A-site cation (La) introduces an effective negative charge which is compensated either by an increase in valence of the B-site cations or the formation of oxygen vacancies (ionic compensation) [34,35]. Zr can perform a valence change like [36] to compensate for the introduced charge imbalance and create couples that act as hopping sites for electrons/holes, i.e., for n-type or p-type conductivity. From Fig 8 it is clear that electrical conductivity increases with an increase in temperature since intrinsic defects are activated by elevated temperature. On the other hand, electrical conductivity also increases with ZrO_2_ since it creates extrinsic defects and produces hoping sites (holes).

On the other hand, Manganese-containing perovskites mostly perform electrical compensation which produces an important defect reaction which is the charge disproportionation reaction by which Mn^3+^ partially disproportionate into Mn^2+^ and Mn^4+^ leading to good electrical conductivity even at lower temperatures.

When analyzing the electrical conductance using the Arrhenius plot log(σ) versus 1/T, linear behavior means that the electrical conductivity is because of the small polaron-hopping mechanism that occurs in the perovskite along the transition metal–oxygen–transition metal chains [37].

### 3.7 Density

**Fig 9:**
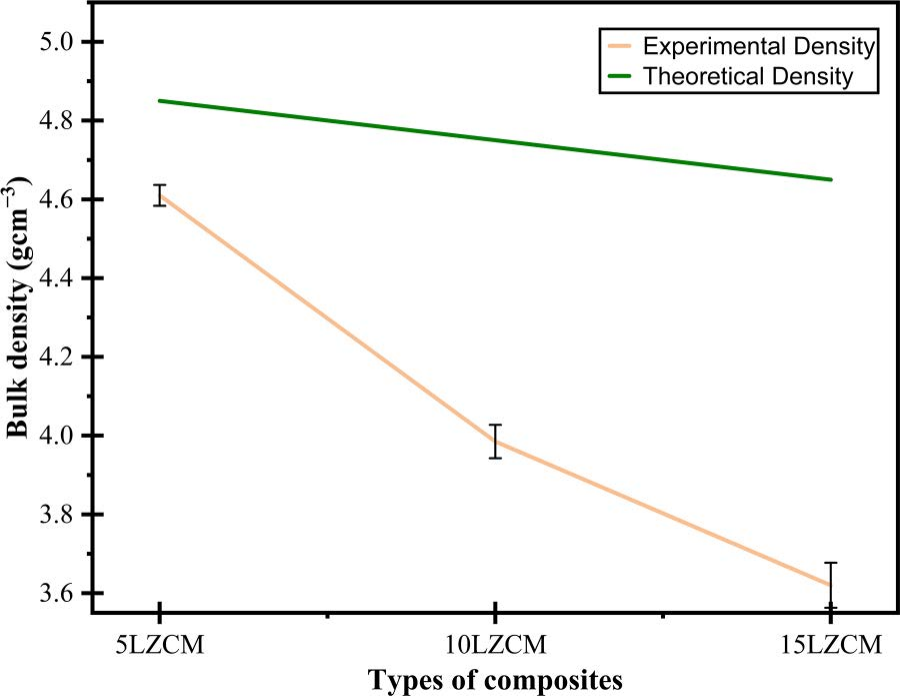
Effect of addition of ZrO_2_ density of perovskite structure.

The effect of ZrO_2_ addition on the composite density is shown in Fig 9 where the sintering temperature of composites was 1200°C. The figure shows that the bulk density decreases with the addition of ZrO_2_ content. Fig 9 also shows the theoretical density of the composites. Since the smaller Zr substitutes the larger La in the perovskite structure the theoretical density increased due to the compactness of the composites. Similarly, the addition of more TiO_2_ resulted in larger particle size and enhanced dissolving into YSZ, which promoted the densification behavior in the Fe_2_O_3_-8YSZ composite [38,39]. Fig 9 shows the relative density of composites, where, sintered density is a function of theoretical density.

### 3.8 Porosity

**Fig 10:**
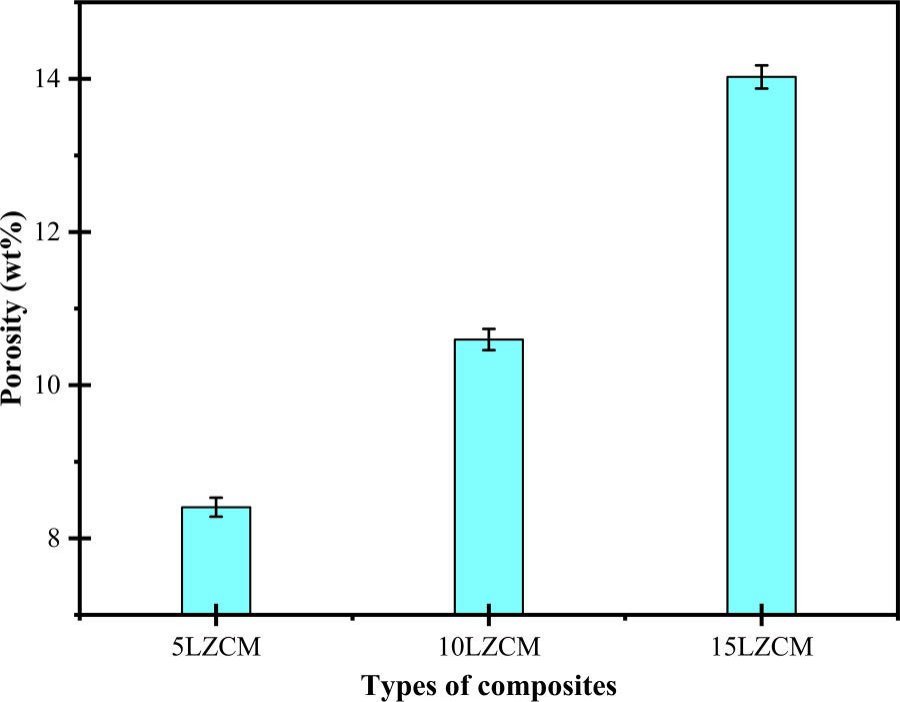
Effect of addition of ZrO_2_ on porosity of perovskite structure.

Fig 10 shows the effect of the addition of ZrO_2_ on the porosity of the composites. The porosity was maximum for 15LZCM. The further addition of ZrO_2_ and MnO_2_ will increase the porosity. But while increasing the amount of doping material will cause more internal defects. At some point, the larger grain increased the inter-granular space which was responsible for increasing the porosity [40,41]. The trend of variation of porosity with the continuous addition of ZrO_2_ was opposite to the trend of density variation. Porosity was minimum at that point where the density was maximum. A similar result was also reported by T. Kinoshita [42].

### 3.9 SEM and EDX

**Fig 11:**
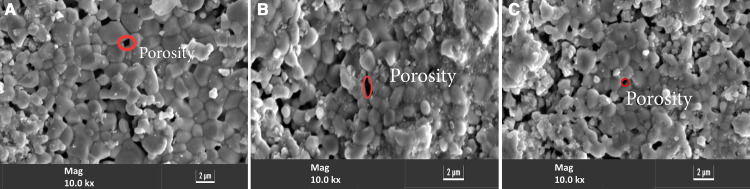
SEM images (a) 5LZCM (b) 10LZCM (c) 15LZCM.

Fig 11 shows the microstructure of La_1−*x*_Zr_*x*_Co_1-y_Mn_y_O_3_ cathode that sintered at 1400°C for 3 hrs. The Scanning Electron Microscope (SEM) images were taken from the surface of the cathode material and showed porous microstructure.

According to the SEM image the cathode material has less porosity and the cathode layer is tightly adhered to the surface. From the porosity test, it was found that for 15LZCM sample is porous and it was also found in SEM analysis. It was also observed that there is no crack at the interface. This type of microstructure shows good electrochemical performance [43].

**Fig 12:**
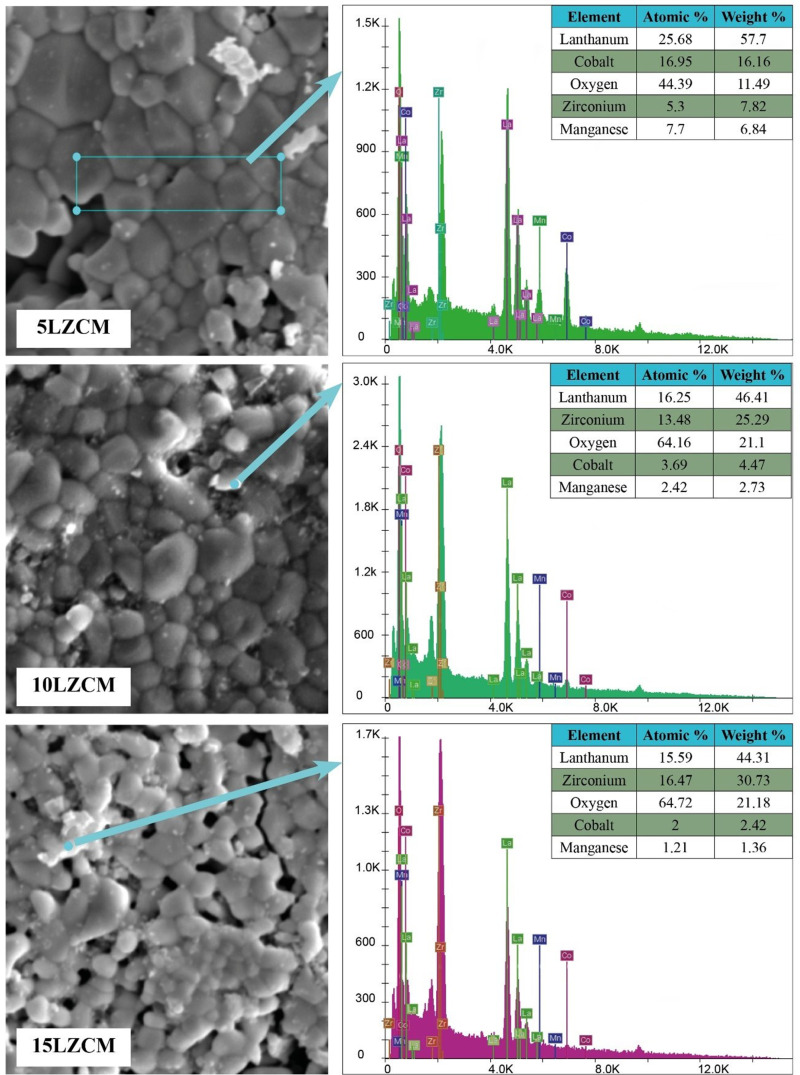
EDX analysis of the fabricated samples.

Fig 12 illustrates the Energy Dispersive X-Ray (EDX) analysis of the samples which sintered at 1400°C. The atomic percentage, weight percentage, and present elements are shown in Fig 12. The analysis results are insufficient to reliably determine the actual composition of the area. Nina et. al conducted EDX for the IT-SOFC cathode materials at 700°C [44].

### 3.10 Atomic force microscopy (AFM)

**Fig 13:**
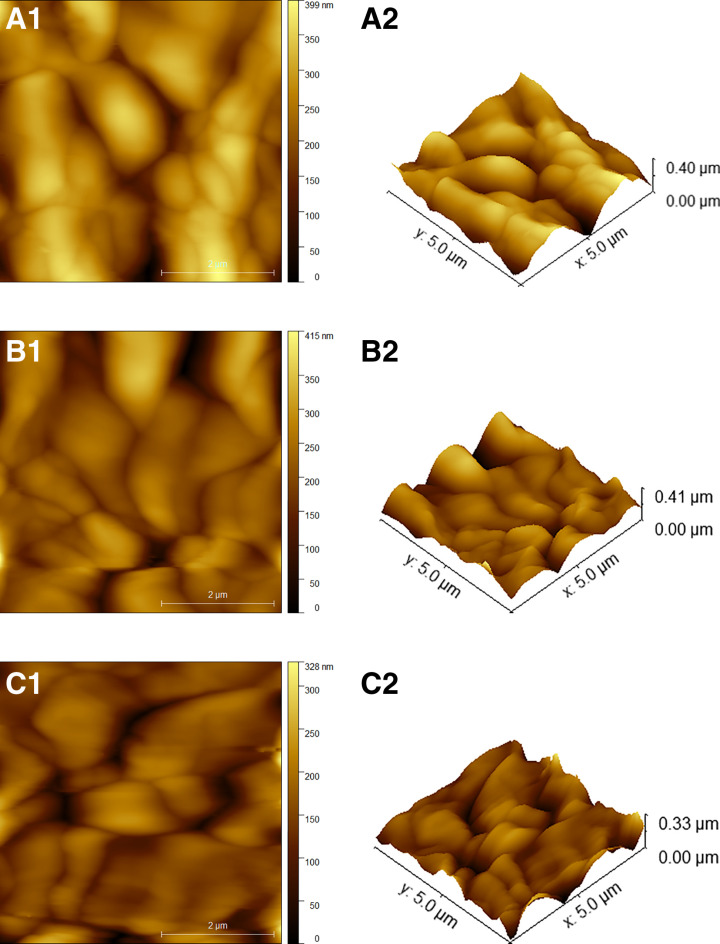
2D and 3D AFM micrographs of fabricated samples (a) 5LZCM (b) 10LZCM (c) 15LZCM.

Fig 13 shows the micrograph of the fabricated composites. AFM images are visualizations of numerical values, enabling quantitative study, with the quality of analysis reliant on the quality of the AFM picture. Table 3 shows about the S_q_ (root mean square roughness), S_a_ (mean roughness), S_p_ (maximum peak), S_v_ (Maximum pit depth), S_z_ (maximum height) of the fabricated samples. The rms roughness was affected due to the addition of ZrO_2_, increasing the ZrO_2_ and decreasing the roughness, the maximum value was found for 5LZCM and minimum for 15LZCM. From the porosity test, it was observed that maximum porosity was obtained for 5LZCM. Therefore, the higher value of maximum pit depth was measured for 5LZCM. The higher value of maximum peak height and maximum height was obtained for 10LZCM. Particle-shaped contact marks describe the electrochemically generated interfaces on in situ-built electrodes under cathodic polarization conditions [45].

**Table 3:** Measured parameters of the fabricated LZCM composites.

Fabricated Composites	RMS roughness (S_q_) nm	Mean roughness (S_a_) nm	Maximum peak height (S_p_) nm	Maximum pit depth (S_v_) nm	Maximum height (S_z_) nm
**5LZCM**	61.46	49.84	155.75	242.82	398.57
**10LZCM**	52.42	39.19	203.98	210.79	414.78
**15LZCM**	37.12	28.12	182.54	145.19	327.74

Table 4 compares the mechanical and physical properties of ZrO_2_-doped composites (5LZCM, 10LZCM, 15LZCM). As ZrO_2_ doping increases, diametral tensile strength, compressive strength, and hardness improve significantly, indicating enhanced mechanical performance. However, density decreases and porosity increases with higher doping levels, reflecting the trade-off between structural compactness and functional enhancement in the composites.

**Table 4:** The comparisons of key properties for different ZrO_2_-doped fabricated composites.

Fabricated Composites	Mechanical Properties			Physical Properties	
	Diametral Tensile Strength (MPa)	Compressive Strength (MPa)	Hardness	Density	Porosity
5LZCM	16.29	58.27	84.5	4.61	8.40
10LZCM	23.68	62.94	96.3	3.98	10.59
15LZCM	24.97	78.56	125.4	3.62	14.02

## Conclusion

The composites were sintered at 1200°C in this research. The ZrO_2_ were varied and MnO_2_ was kept constant. The ZrO_2_ content varied from 5 wt% to 15 wt% as A site substitution and MnO_2_ content kept constant at 30 wt% as B site substitution.

The key findings of the study are:

- The hardness increased with the increment of addition of doping material as ZrO_2_ substitute A site cation and MnO_2_ substitute B site cation of perovskite structure and the hardness increases.
- The diametral tensile strength is increased from 16.5 MPa to 26 MPa as doping concentration increases. The XRD investigation showed that the addition of ZrO_2_ caused a change in the structure of the perovskite. This resulted in a monoclinic crystal system with the space group P 1 21/c1.
- The perovskite oxide exhibited a porous morphology as seen by SEM research.
- AFM revealed that the presence of ZrO_2_ decreased the surface roughness.

The findings indicate that ZrO_2_ doping improves the structural and functional features of La_1-*x*_Zr_*x*_Co_1−y_Mn_y_O_3_ composites, making them suitable for high-performance applications.

## Supporting information

S1 File Supporting Dataset. (ZIP)

## References

[1] Subardi A, Indra A, Setiawan J, Fu Y-P. Structural and Electrochemical Analysis of SmBa0.8Sr0.2Co2O5+δ Cathode Oxide for IT-SOFCs. IJIE. 2023;15(1). doi: 10.30880/ijie.2023.15.01.015

[2] Stambouli AB, Traversa E, Stambouli A. Solid oxide fuel cells (SOFCs): a review of an environmentally clean and efficient source of energy. 2002. [Online]. Available from: www.elsevier.com/locate/rser

[3] Hassan MS. Electrochemical performance of Pr0.6Sr0.4Fe0.8Co0.2O3−δ as potential cathode material for IT-SOFC. Mater Tech Rep. 2024;2(1):483. doi: 10.59400/mtr.v2i1.483

[4] Ashrafi H, Pourmahmoud N, Mirzaee I, Ahmadi N. Performance improvement of proton‐exchange membrane fuel cells through different gas injection channel geometries. Int J Energy Res. 2022;46(7):8781–92.

[5] Bose D. Fuel Cells: The Fuel for Tomorrow. Journal of Energy Research and Environmental Technology. 2023;2(2):71–5.

[6] Tan L, Dong X, Gong Z, Wang M. Analysis on energy efficiency and CO2 emission reduction of an SOFC-based energy system served public buildings with large interior zones. Energy. 2018;165:1106–18. doi: 10.1016/j.energy.2018.10.054

[7] Li L, Kong Z, Yao B, Yang H, Gao Z, Xu L, et al. An efficient and durable perovskite electrocatalyst for oxygen reduction in solid oxide fuel cells. Chemical Engineering Journal. 2020;396:125237.

[8] Song X, Le S, Zhu X, Qin L, Luo Y, Li Y, et al. High performance BaFe1− xBixO3− δ as cobalt-free cathodes for intermediate temperature solid oxide fuel cells. International Journal of Hydrogen Energy. 2017;42(24):15808–17.

[9] Subardi A, Indra A, Setiawan J, Fu Y-P. Structural and Electrochemical Analysis of SmBa0.8Sr0.2Co2O5+δ Cathode Oxide for IT-SOFCs. IJIE. 2023;15(1). doi: 10.30880/ijie.2023.15.01.015

[10] Yu X, Long W, Jin F, He T. Cobalt-free perovskite cathode materials SrFe1−xTixO3−δ and performance optimization for intermediate-temperature solid oxide fuel cells. Electrochimica Acta. 2014;123:426–34. doi: 10.1016/j.electacta.2014.01.020

[11] Omeiza LA, Mamudu U, Subramanian Y, Dhanasekaran A, Rahman MM, Bakar SA, et al. Structure and electrochemical characterization of Nd0.5Ba0.5Zr0.8Fe0.2O3 δ cobalt-free cathode material for intermediate-temperature solid oxide fuel cells: An experimental investigation. Johnson Matthey Technology Review. 2027.

[12] Mostafavi E, Ataie A, Ahmadzadeh M, Palizdar M, Comyn TP, Bell AJ. Synthesis of nano-structured Bi1−xBaxFeO3 ceramics with enhanced magnetic and electrical properties. Materials Chemistry and Physics. 2015;162:106–12. doi: 10.1016/j.matchemphys.2015.05.017

[13] Sallam OI, Rammah YS, Nabil IM, El-Seidy AMA. Enhanced optical and structural traits of irradiated lead borate glasses via Ce3+ and Dy3+ ions with studying Radiation shielding performance. Sci Rep. 2024;14(1):24478. doi: 10.1038/s41598-024-73892-w 39424847 PMC11489820

[14] Nabil IM, El-Seidy AMA, Mosleh AT, Zahran HY, Zyoud SH, Yahia IS. Influence of low copper oxide additives on B2O3-Li2O-Na2O-CaO-SrO-As2O3 glasses: a physical, structural, and radiological study. Journal of Materials Science: Materials in Electronics. 2024;35(19):1–20. doi: 10.1007/S10854-024-12891-Z/METRICS

[15] Shisode MV, Bhoyar DN, Khirade PP, Jadhav KM. Structural, Microstructural, Magnetic, and Ferroelectric Properties of Ba 2 + -Doped BiFeO3 Nanocrystalline Multifferroic Material. J Supercond Nov Magn. 2017;31(8):2501–9. doi: 10.1007/s10948-017-4515-5

[16] Caro J, Wang HH, Tablet C, Kleinert A, Feldhoff A, Schiestel T, et al. Evaluation of perovskites in hollow fibre and disk geometry in catalytic membrane reactors and in oxygen separators. Catalysis Today. 2006;118(1–2):128–35.

[17] Li Z, Ge Y, Xiao Y, Du M, Yang F, Ma Y, Gao D. High Entropy Designed (Sr0. 2ba0. 2bi0. 2la0. 2pr0. 2) Feo3 It-Sofc Cathode Fabrication and Performance Investigation. Available from: SSRN 4662104.

[18] Kida T, Yamasaki A, Watanabe K, Yamazoe N, Shimanoe K. Oxygen-permeable membranes based on partially B-site substituted BaFe1−yMyO3−δ (M=Cu or Ni). Journal of Solid State Chemistry. 2010;183(10):2426–31. doi: 10.1016/j.jssc.2010.08.002

[19] Liu X, Zhao H, Yang J, Li Y, Chen T, Lu X, et al. Lattice characteristics, structure stability and oxygen permeability of BaFe1−Y O3− ceramic membranes. Journal of Membrane Science. 2011;383(1–2):235–40. doi: 10.1016/j.memsci.2011.08.059

[20] Bongio EV, Black H, Raszewski FC, Edwards D, McConville CJ, Amarakoon VR. Microstructural and high-temperature electrical characterization of La 1−x Sr x FeO 3−δ.

[21] Petric A, Huang P, Tietz F. Evaluation of La-Sr-Co-Fe-O perovskites for solid oxide fuel cells and gas separation membranes. 2000.

[22] Kostogloudis GC, Vasilakos N, F t i k o s C. Preparation and characterization of Pr 1 _,Sr,MnO~ 6 (x = 0, O-15, O-3, 0.4, 0.5) as a potential SOFC cathode material operating at intermediate temperatures (500-700°C). 1997.

[23] Huang YH, Karppinen M, Yamauchi H, Goodenough JB. Systematic studies on effects of cationic ordering on structural and magnetic properties inSr2FeMoO6. Phys Rev B. 2006;73(10). doi: 10.1103/physrevb.73.104408

[24] Richter J, Holtappels P, Graule T, Nakamura T, Gauckler LJ. Materials design for perovskite SOFC cathodes. Monatsh Chem. 2009;140(9):985–99. doi: 10.1007/s00706-009-0153-3

[25] Teraoka Y, Nobunaga T, Okamoto K, Miura N, Yamazoe N. SOLID STATE IOlllCS Influence of constituent metal cations in substituted LaCoO3 on mixed conductivity and oxygen permeability. 1991.

[26] Dong F, Ni M, Chen Y, Chen D, Tadé MO, Shao Z. Structural and oxygen-transport studies of double perovskites PrBa1−xCo2O5+δ(x = 0.00, 0.05, and 0.10) toward their application as superior oxygen reduction electrodes. J Mater Chem A. 2014;2(48):20520–9. doi: 10.1039/c4ta04372c

[27] Ling Y, Zhang X, Wang Z, Wang S, Zhao L, Liu X, et al. Potentiality of cobalt-free perovskite Ba0.5Sr0.5Fe0.9Mo0.1O3−δ as a single-phase cathode for intermediate-to-low-temperature solid oxide fuel cells. International Journal of Hydrogen Energy. 2013;38(33):14323–8.

[28] Islam MdR, Islam MdS, Zubair MA, Usama HM, Azam MdS, Sharif A. Evidence of superparamagnetism and improved electrical properties in Ba and Ta co-doped BiFeO3 ceramics. Journal of Alloys and Compounds. 2018;735:2584–96. doi: 10.1016/j.jallcom.2017.11.323

[29] Al-Amin Md, Mumu HT, Sarker S, Alam MdZ, Gafur MA. Effects of sintering temperature and zirconia content on the mechanical and microstructural properties of MgO, TiO2 and CeO2 doped alumina–zirconia (ZTA) ceramic. J Korean Ceram Soc. 2022;60(1):141–54. doi: 10.1007/s43207-022-00194-0

[30] Vivekananthan M, Ahilan C, Sakthivelu S, Saravanakumar M. A primary study of density and compressive strength of the silicon nitride and titanium nitride ceramic composite. Materials Today: Proceedings. 2020;33:2741–5. doi: 10.1016/j.matpr.2020.01.570

[31] de Salazar JMG, Barrena MI, Morales G, Matesanz L, Merino N. Compression strength and wear resistance of ceramic foams–polymer composites. Mater Lett. 2006;60(13–14):1687–92.

[32] Ma W, Mack DE, Vaßen R, Stöver D. Perovskite‐Type Strontium Zirconate as a New Material for Thermal Barrier Coatings. Journal of the American Ceramic Society. 2008;91(8):2630–5. doi: 10.1111/j.1551-2916.2008.02472.x

[33] Lu P, Zheng Y, Cheng J, Guo D. Effect of La2O3 addition on crystallization and properties of Li2O–Al2O3–SiO2 glass-ceramics. Ceramics International. 2013;39(7):8207–12. doi: 10.1016/j.ceramint.2013.04.004

[34] Bard J, Faulkner LR. Electrochemical properties of mixed conducting perovskites (M = Sr, Ba, Ca). Seoul National University Press. 1996.

[35] Huang X, Liu J, Lu Z, Liu W, Pei L, He T, et al. Properties of nonstoichiometric Pr0.6−xSr0.4MnO3 as the cathodes of SOFCs. Solid State Ionics. 2000;130(3–4):195–201.

[36] Inoue IH. Electrostatic carrier doping to perovskite transition-metal oxides. Semicond Sci Technol. 2005;20(4):S112–20. doi: 10.1088/0268-1242/20/4/013

[37] Huang X, Pei L, Liu Z, Lu Z, Sui Y, Qian Z, et al. A study on PrMnO3-based perovskite oxides used in SOFC cathodes. Journal of Alloys and Compounds. 2002;345(1–2):265–70.

[38] Luo P, Zhang J, You Z, Ran X, Liu Y, Li S, et al. Effect of TiO2 content on the microstructure and mechanical and wear properties of yttria-stabilized zirconia ceramics prepared by pressureless sintering. Materials Research Express. 2020;6(12):125211.

[39] Guo F, Xiao P. Effect of Fe2O3 doping on sintering of yttria-stabilized zirconia. J Eur Ceram Soc. 2012;32(16):4157–64.

[40] Prakash Nautiyal O, Bhatt SC, Nautiyal OP. Preparation and Characterization of Lithium Doped Silver Niobate Perovskite System. 2011. [Online]. Available from: http://journal.sapub.org/materials

[41] Huang A, Yao K, Wang J. Conducting perovskite LaNi0.6Co0.4O3 ceramics with glass additions. J Electroceram. 2006;16(4):313–9. doi: 10.1007/s10832-006-9871-7

[42] Kinoshita T, Arastoo A, Maruko H, Adachi M. Synthesis of Porous Particles of SOFC Anode and Cathode Materials by Citric Acid-Addition Ultrasonic Spray Pyrolysis (CA-USP). Aerosol Science and Technology. 2014;48(10):1089–98. doi: 10.1080/02786826.2014.958130

[43] Li H, Sun LP, Li Q, Xia T, Zhao H, Huo LH, et al. Electrochemical performance of double perovskite Pr2NiMnO6 as a potential IT-SOFC cathode. International Journal of Hydrogen Energy. 2015;40(37):12761–9.

[44] Schrödl N, Bucher E, Egger A, Kreiml P, Teichert C, Höschen T, et al. Long-term stability of the IT-SOFC cathode materials La0.6Sr0.4CoO3−δ and La2NiO4 δ against combined chromium and silicon poisoning. Solid State Ionics. 2015;276:62–71.

[45] Jiang SP. Thermally and Electrochemically Induced Electrode/Electrolyte Interfaces in Solid Oxide Fuel Cells: An AFM and EIS Study. J Electrochem Soc. 2015;162(10):F1119–28. doi: 10.1149/2.0111510jes

