# Supplementary material for: Influence of ZrO2 content on the mechanical, electrical, and microstructural characteristics of La1-xZrxCo1−yMnyO3 perovskites for IT-SOFC cathodes
Source: PLoS One. 2025 Jun 4;20(6):e0320562. doi: 10.1371/journal.pone.0320562 (PMC12136471; doi:10.1371/journal.pone.0320562)
Supplement: S1 File — (ZIP) [file pone.0320562.s001.zip › Supporting Dataset IT-SOFC/SEM-EDX/EDX_10LZCM.pdf]

TESCAN EDS Report

Created 2024-01-28 17:09

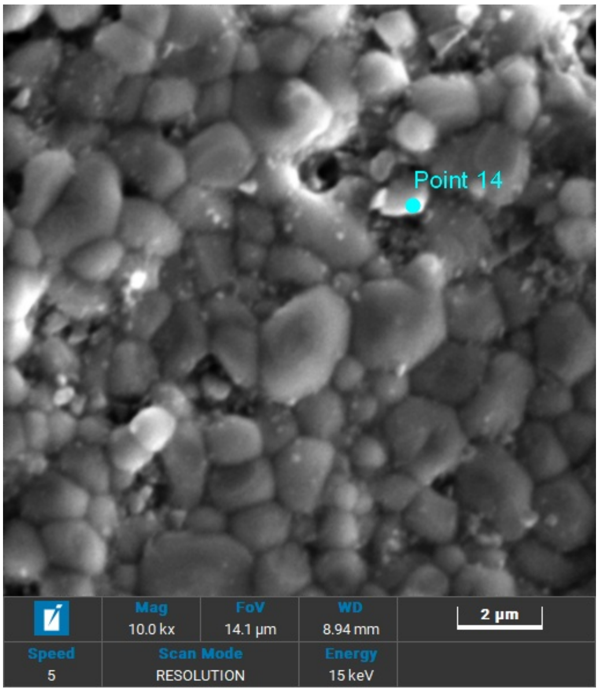

Type	Point
Profile	Rate
Mode	Continual
Counts	297 863
Real Time	151.368
Live Time	151.003
Dead Time	0 %
Landing Energy	15 keV
Beam Current	300 pA
Coating Element	Gold

Spectrum

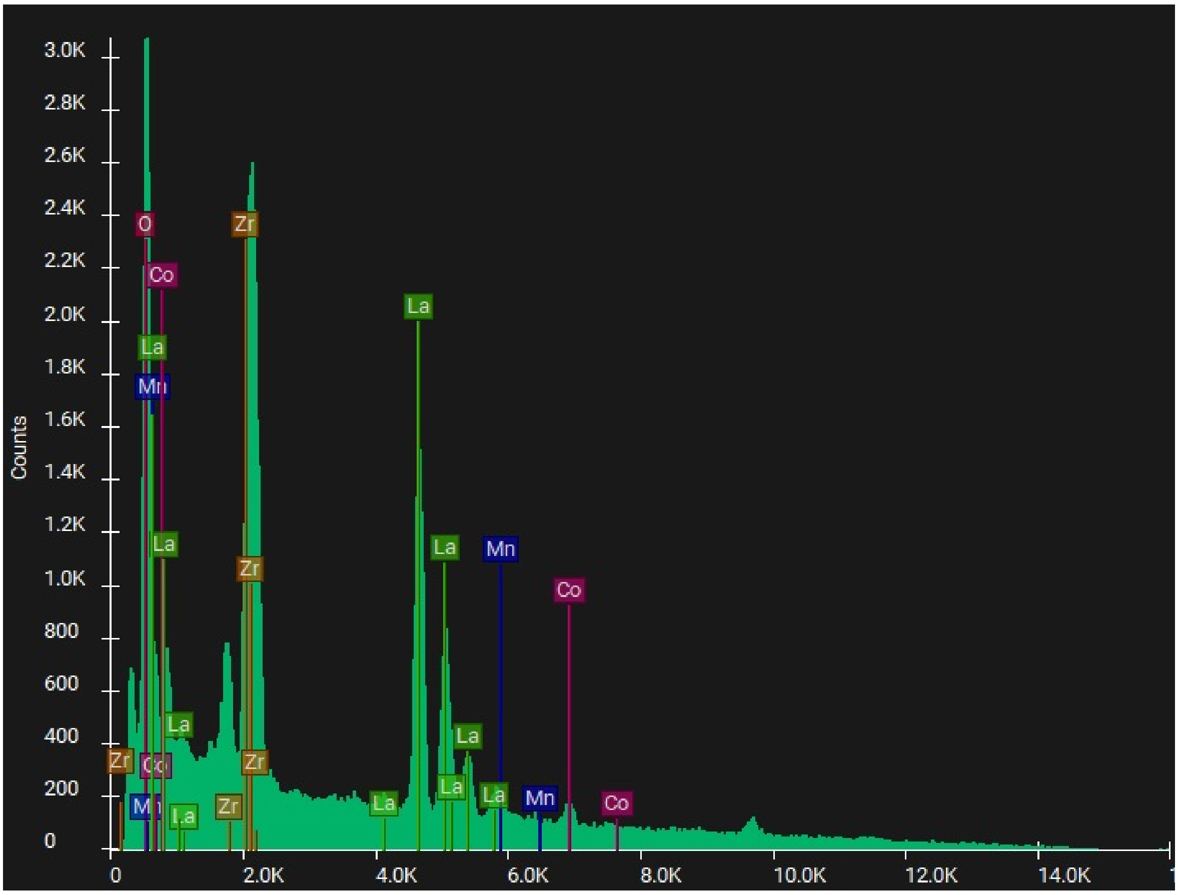

Quantity analysis

Element	Atomic %	Weight %
Cobalt	3.69	4.47
Lanthanum	16.25	46.41
Manganese	2.42	2.73
Oxygen	64.16	21.10
Zirconium	13.48	25.29

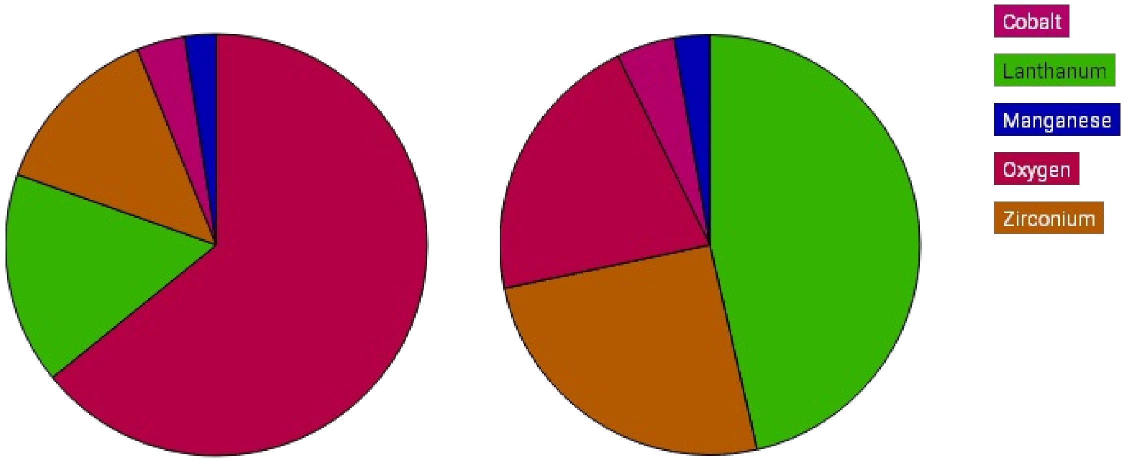

Atomic fraction Weight fraction
