# Supplementary material for: Influence of ZrO2 content on the mechanical, electrical, and microstructural characteristics of La1-xZrxCo1−yMnyO3 perovskites for IT-SOFC cathodes
Source: PLoS One. 2025 Jun 4;20(6):e0320562. doi: 10.1371/journal.pone.0320562 (PMC12136471; doi:10.1371/journal.pone.0320562)
Supplement: S1 File — (ZIP) [file pone.0320562.s001.zip › Supporting Dataset IT-SOFC/SEM-EDX/EDX_15LZCM.pdf]

# TESCAN EDS Report

Created 2024-01-29 12:26

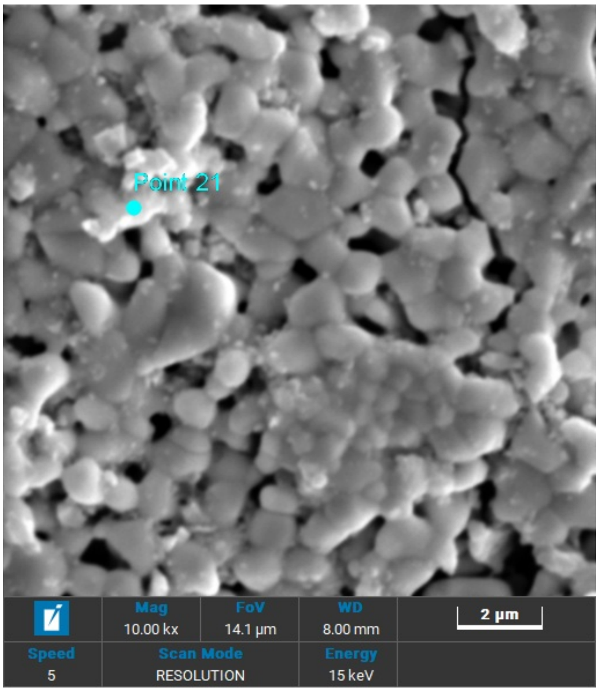


| | |
|---|---|
| Type | Point |
| Profile | Rate |
| Mode | Continual |
| Counts | 173 314 |
| Real Time | 85.0677 |
| Live Time | 84.8556 |
| Dead Time | 0 % |
| Landing Energy | 15 keV |
| Beam Current | 300 pA |
| Coating Element | Gold |

## Spectrum

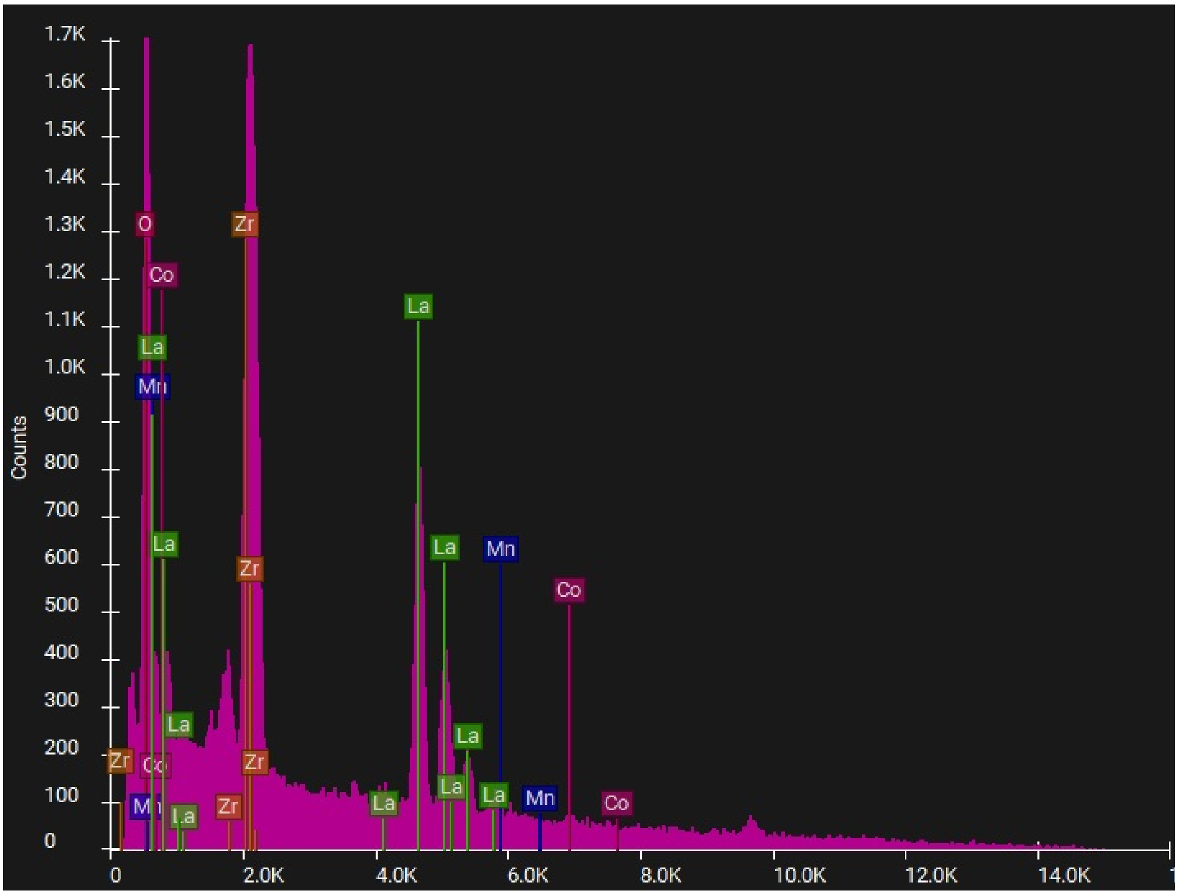


## Quantity analysis

| Element | Atomic % | Weight % |
|---|---|---|
| Cobalt | 2.00 | 2.42 |
| Lanthanum | 15.59 | 44.31 |
| Manganese | 1.21 | 1.36 |
| Oxygen | 64.72 | 21.18 |
| Zirconium | 16.47 | 30.73 |

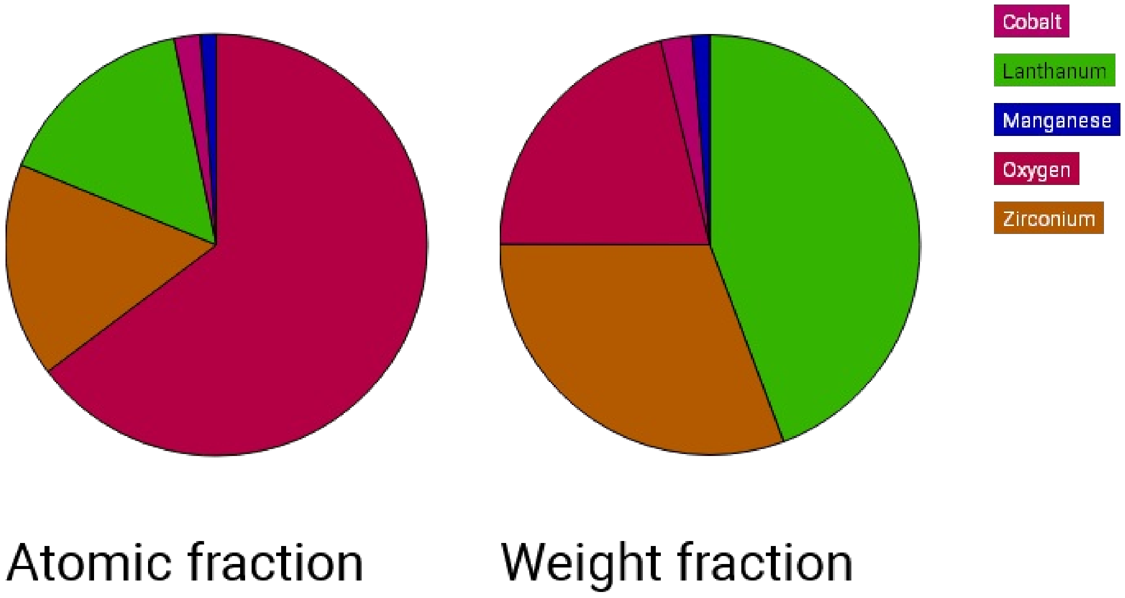
Cobalt
Lanthanum
Manganese
Oxygen
Zirconium
Atomic fraction
Weight fraction
