# Supplementary material for: Influence of ZrO2 content on the mechanical, electrical, and microstructural characteristics of La1-xZrxCo1−yMnyO3 perovskites for IT-SOFC cathodes
Source: PLoS One. 2025 Jun 4;20(6):e0320562. doi: 10.1371/journal.pone.0320562 (PMC12136471; doi:10.1371/journal.pone.0320562)
Supplement: S1 File — (ZIP) [file pone.0320562.s001.zip › Supporting Dataset IT-SOFC/SEM-EDX/EDX_5LZCM.pdf]

TESCAN EDS Report

Created 2024-01-25 17:09

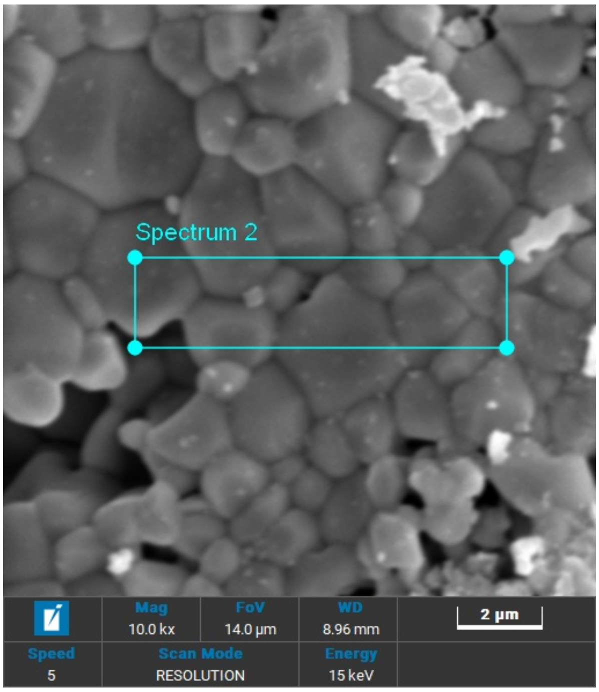

Type	Area
Profile	Rate
Mode	Continual
Counts	175 003
Real Time	116.185
Live Time	115.968
Dead Time	0 %
Landing Energy	15 keV
Beam Current	250 pA
Coating Element	Gold

Spectrum

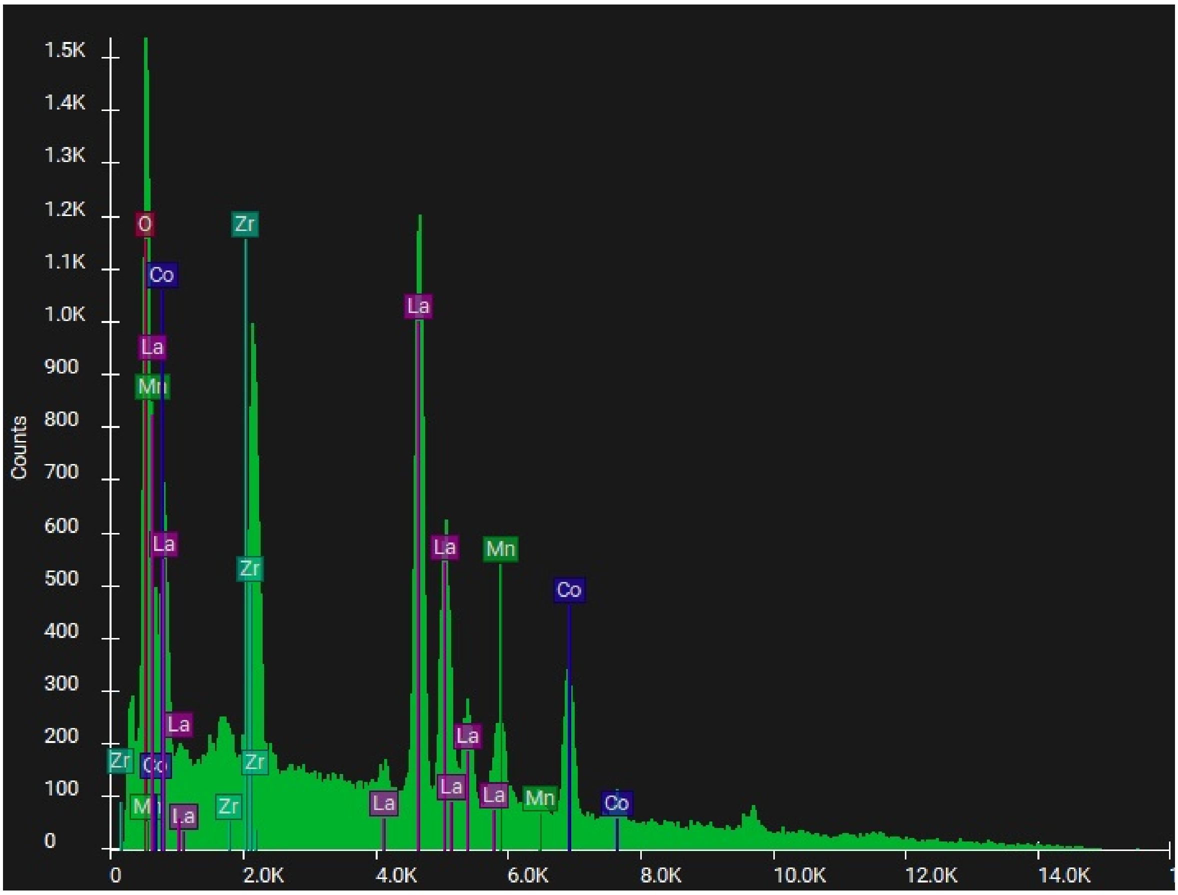

Quantity analysis

Element	Atomic %	Weight %
Cobalt	16.95	16.16
Lanthanum	25.68	57.70
Manganese	7.70	6.84
Oxygen	44.39	11.49
Zirconium	5.30	7.82

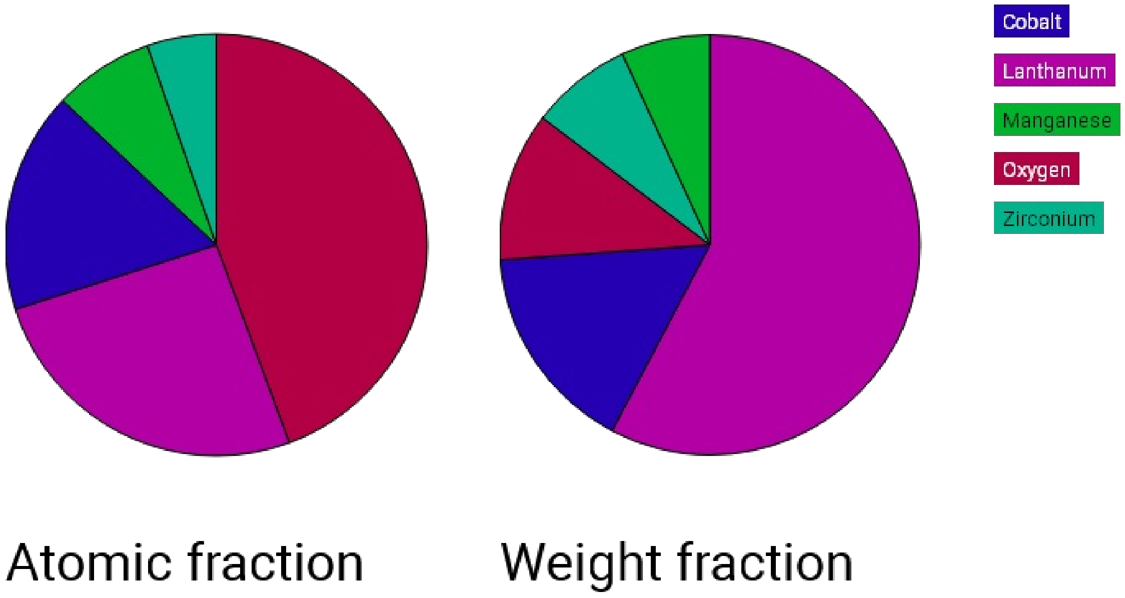
Cobalt
Lanthanum
Manganese
Oxygen
Zirconium
Atomic fraction
Weight fraction
