# Supplementary figures and images for: Influence of ZrO2 content on the mechanical, electrical, and microstructural characteristics of La1-xZrxCo1−yMnyO3 perovskites for IT-SOFC cathodes
Source: PLoS One. 2025 Jun 4;20(6):e0320562. doi: 10.1371/journal.pone.0320562 (PMC12136471; doi:10.1371/journal.pone.0320562)

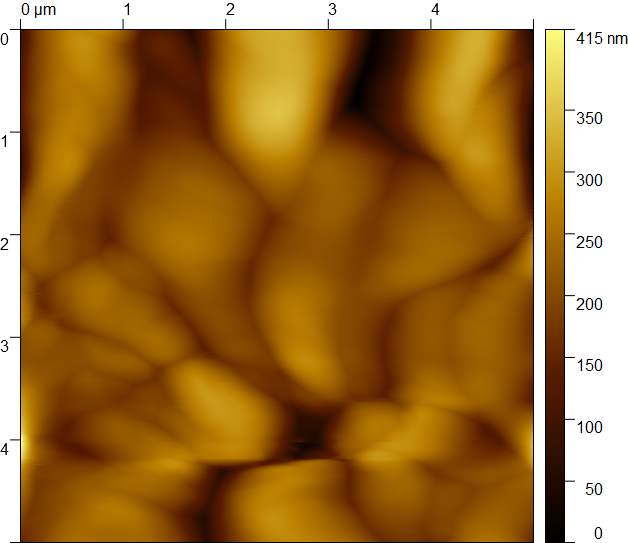

Supplement: S1 File — (ZIP) [file pone.0320562.s001.zip › Supporting Dataset IT-SOFC/AFM/10LZCM/10LZCM 2D.tiff]

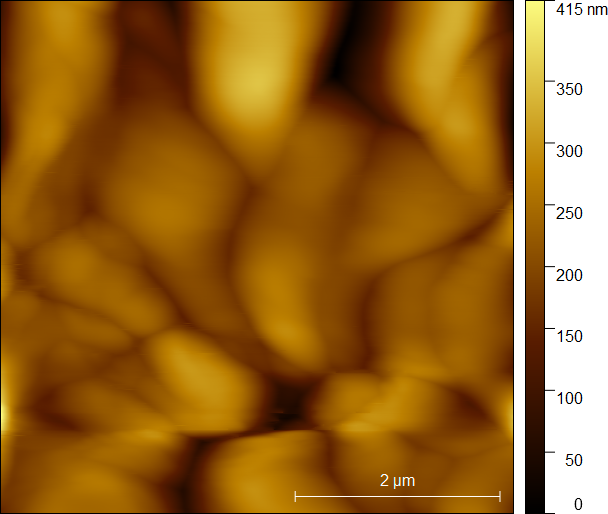

Supplement: S1 File — (ZIP) [file pone.0320562.s001.zip › Supporting Dataset IT-SOFC/AFM/10LZCM/10LZCM 2D-.tiff]

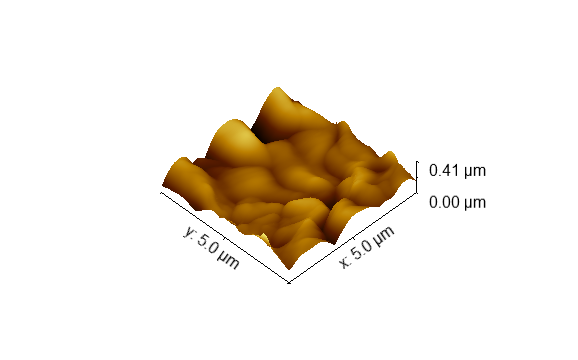

Supplement: S1 File — (ZIP) [file pone.0320562.s001.zip › Supporting Dataset IT-SOFC/AFM/10LZCM/10LZCM 3D.tiff]

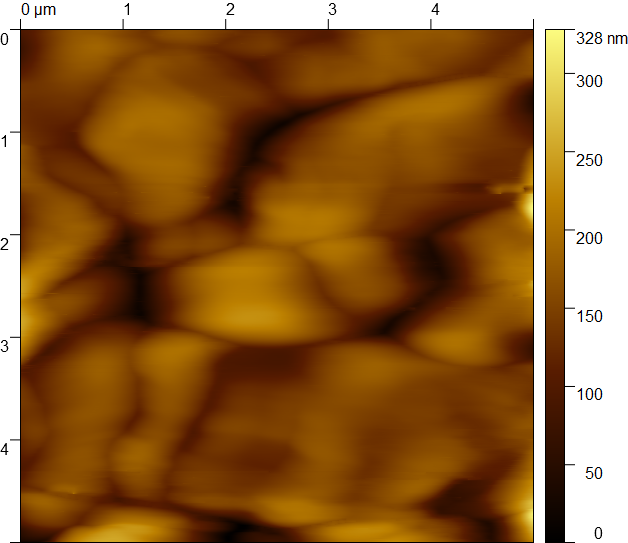

Supplement: S1 File — (ZIP) [file pone.0320562.s001.zip › Supporting Dataset IT-SOFC/AFM/15LZCM/15LZCM 2D.tiff]

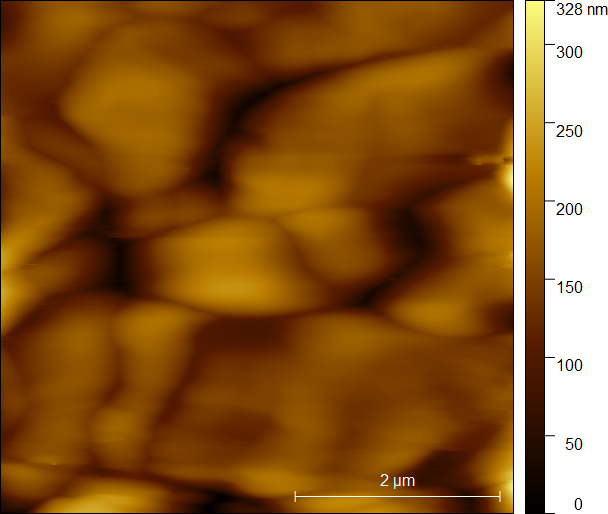

Supplement: S1 File — (ZIP) [file pone.0320562.s001.zip › Supporting Dataset IT-SOFC/AFM/15LZCM/15LZCM 2D-.tiff]

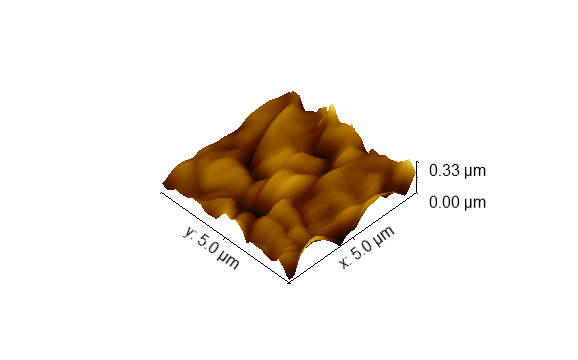

Supplement: S1 File — (ZIP) [file pone.0320562.s001.zip › Supporting Dataset IT-SOFC/AFM/15LZCM/15LZCM 3D.tiff]

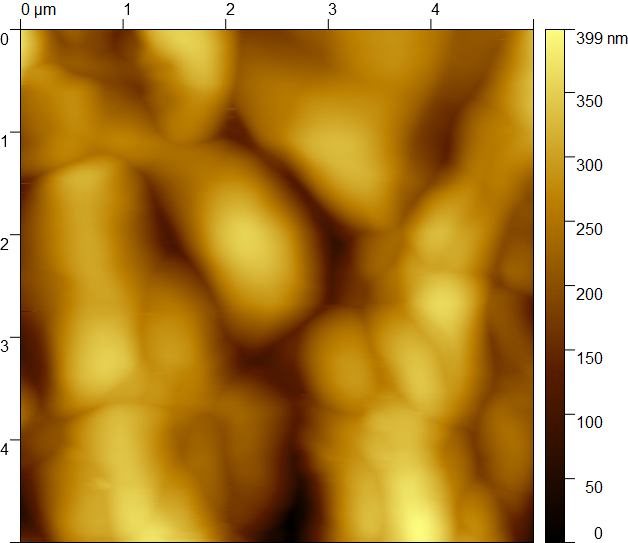

Supplement: S1 File — (ZIP) [file pone.0320562.s001.zip › Supporting Dataset IT-SOFC/AFM/5LZCM/5LZCM 2D.tiff]

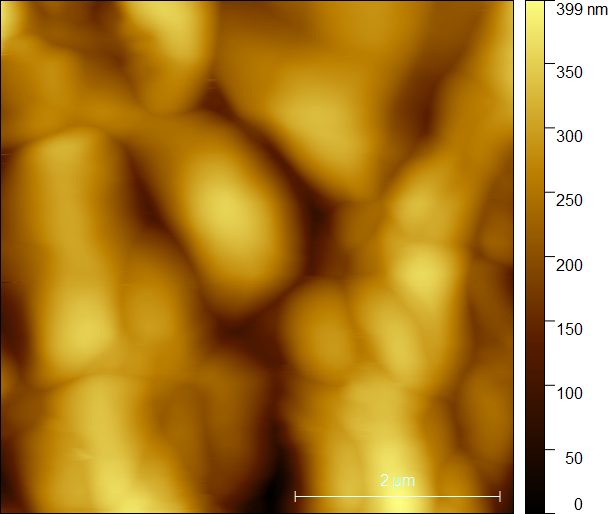

Supplement: S1 File — (ZIP) [file pone.0320562.s001.zip › Supporting Dataset IT-SOFC/AFM/5LZCM/5LZCM 2D-.tiff]

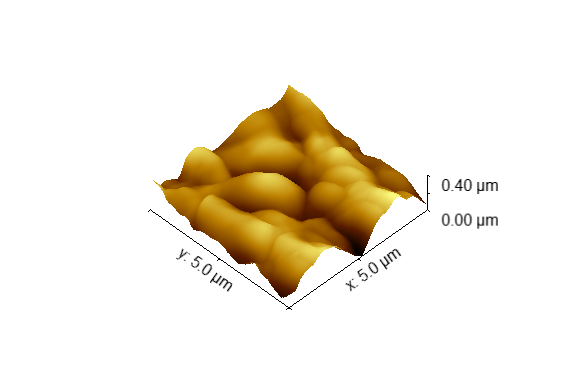

Supplement: S1 File — (ZIP) [file pone.0320562.s001.zip › Supporting Dataset IT-SOFC/AFM/5LZCM/5LZCM 3D.tiff]

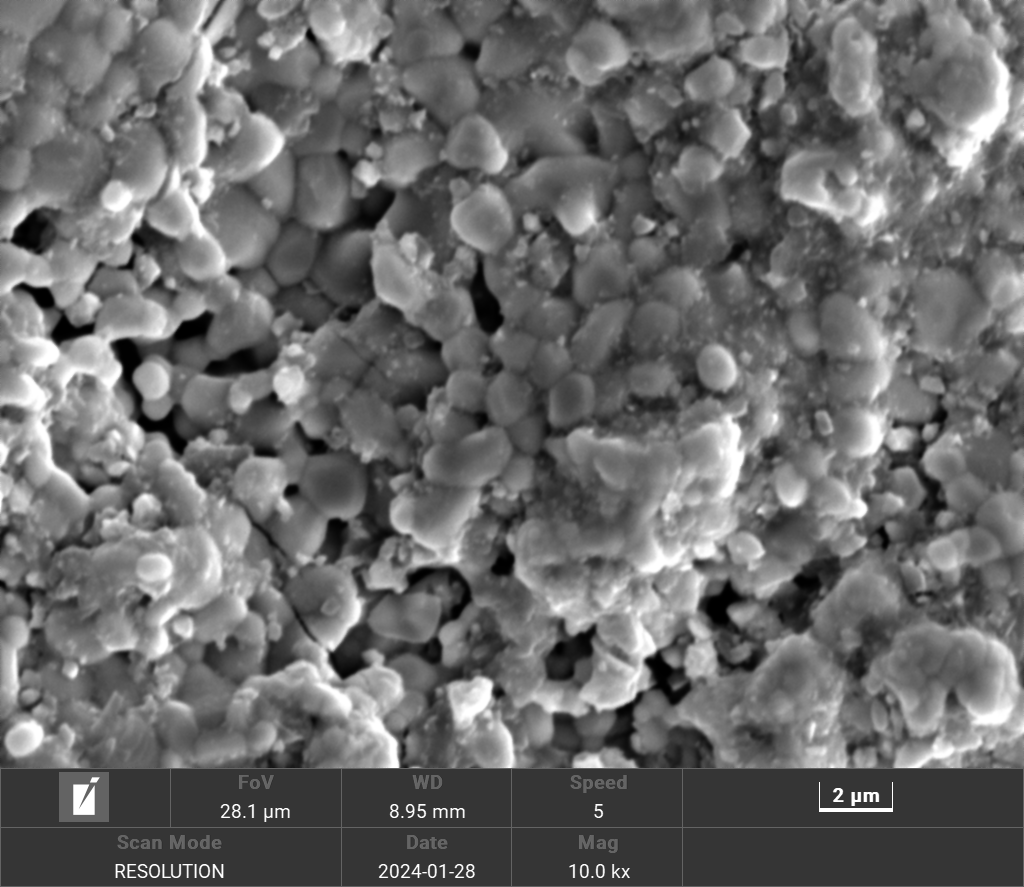

Supplement: S1 File — (ZIP) [file pone.0320562.s001.zip › Supporting Dataset IT-SOFC/SEM-EDX/SEM_10LZCM.tif]

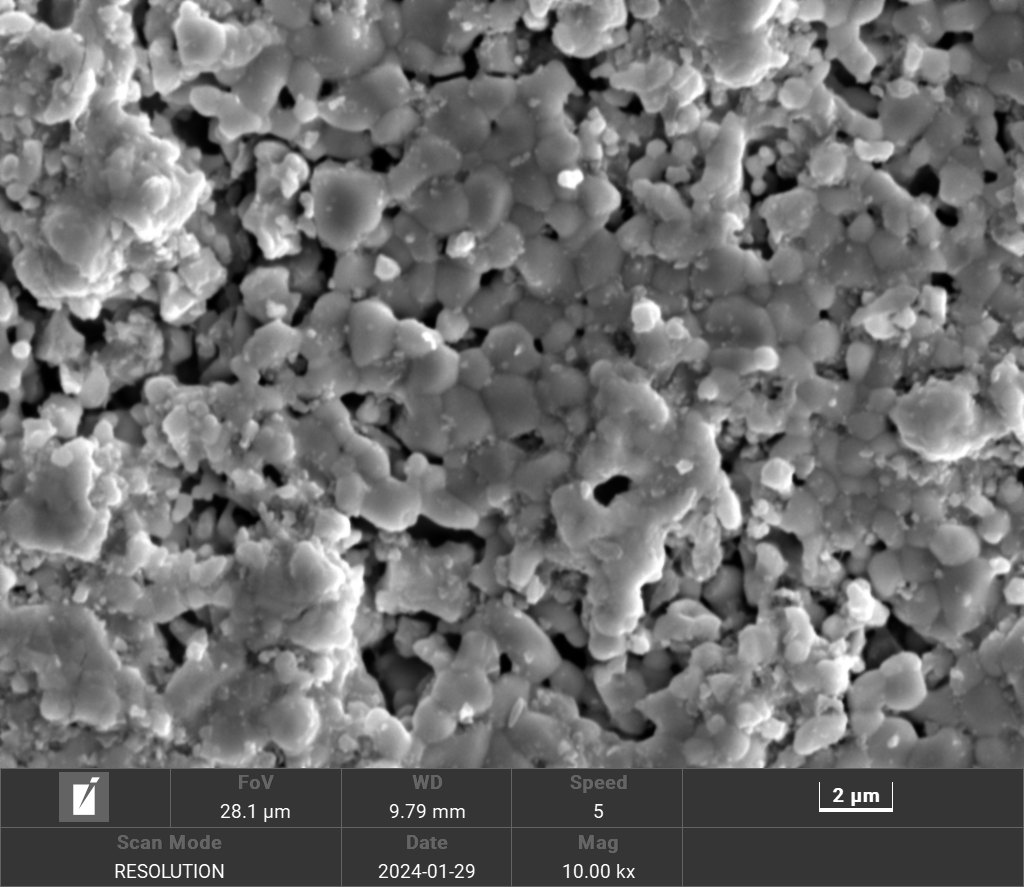

Supplement: S1 File — (ZIP) [file pone.0320562.s001.zip › Supporting Dataset IT-SOFC/SEM-EDX/SEM_15LZCM.tif]

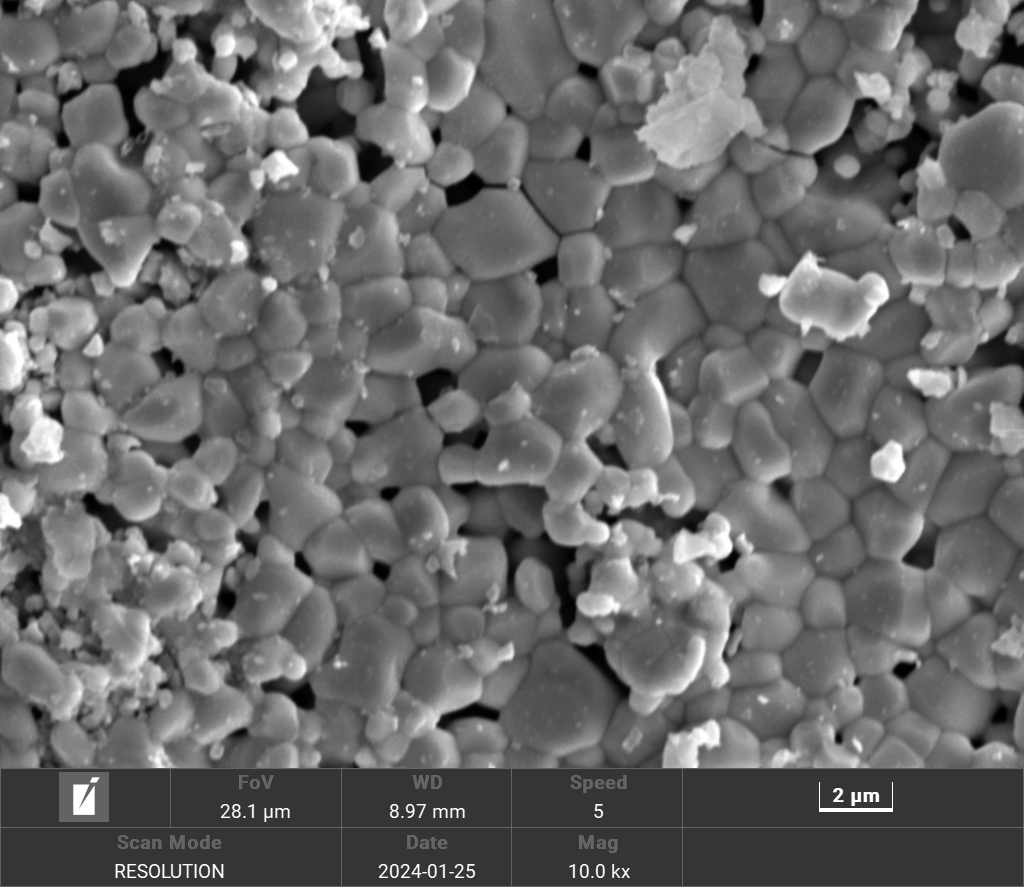

Supplement: S1 File — (ZIP) [file pone.0320562.s001.zip › Supporting Dataset IT-SOFC/SEM-EDX/SEM_5LZCM.tif]
